# Hopelessness, Suicidality, and Co-Occurring Substance Use among Adolescent Hallucinogen Users—A National Survey Study

**DOI:** 10.3390/children9121906

**Published:** 2022-12-05

**Authors:** Saral Desai, Vidisha Jain, Sona Xavier, Wei Du

**Affiliations:** 1 Department of Psychiatry, Tower Health/Phoenixville Hospital, Phoenixville, PA 19460, USA; 2 Department of Pediatrics, Jawaharlal Nehru Medical College, Ajmer 305001, Rajasthan, India; 3 Department of Psychiatry, Father Muller Medical College, Mangaluru 575002, Karnataka, India; 4 Academic Affairs, Tower Health, West Reading, PA 19611, USA; 5 Department of Psychiatry, Drexel University College of Medicine, Philadelphia, PA 19102, USA

**Keywords:** hallucinogens, hopelessness, suicidality, adolescents

## Abstract

(1) Objectives: Hallucinogens are being explored as a potential treatment of psychiatric disorders. Micro dosing of illicitly purchased hallucinogen drugs is on the rise despite conclusive benefits. We aimed to evaluate the prevalence and odds of hopelessness, suicidality, and co-occurring substance use among adolescent hallucinogen users. (2) Methods: We performed a retrospective analysis of the Centers for Disease Control and Prevention’s Youth Risk Behavior Surveillance System (YRBSS) 2001–2019 data that nationally represents school-going US adolescents. We identified hallucinogen use based on the survey questions, exploring the use of hallucinogens (LSD, PCP, mescaline, and mushrooms). (3) Results: Out of a total of 125,550 respondents, 8.4% reported using hallucinogens. Overall, the trend of hallucinogen use decreased from 13.3% (2001) to 7.0% (2019) (pTrend < 0.0001). Hallucinogen users were at high odds of feeling sad and hopeless (aOR: 1.40; 95%CI: 1.21–1.61; *p* < 0.0001), considering suicide (aOR: 1.36; 95%CI: 1.08–1.70; *p* = 0.009), and planning suicide (aOR: 1.49; 95%CI: 1.19–1.86; *p* = 0.001). Additionally, adolescent hallucinogen users had a higher prevalence of alcohol, cigarette, e-cigarette, marijuana, synthetic marijuana, inhalants, heroin, cocaine, methamphetamine, and ecstasy use. (4) Conclusions: The overall trend of hallucinogen use decreased among school-going American adolescents. We found a high prevalence of co-occurring substance use among hallucinogen users. We found that hallucinogen users were at high odds of feeling sad, hopeless, and considering and planning suicide. Further research is needed to explore the effects of recreational hallucinogen use among the adolescent population.

## 1. Introduction

As early as the 1960s, hallucinogens had been categorized into two categories—(1) the serotonergic classic hallucinogens or psychedelics and (2) dissociative anesthetics [1]. Naturally occurring hallucinogens played a crucial role in philosophy and religion in many cultures [2]. Despite promising results seen in the 1950s and 1960s in psychiatry and neuroscience, the association of classic psychedelics with the counterculture and their non-medical use led to the ceasing of psychedelic use in the 1970s for human research [3]. Recently, decriminalization of organic hallucinogens and psilocybin has taken place in a few US cities and the state of Oregon [4].

Hallucinogens’ research has increased over the past decade, mainly psilocybin-related research [5]. Multiple studies have found that hallucinogen use in a controlled setting was associated with a decrease in psychological distress in cancer patients [6,7,8], decreased depressive symptoms in treatment-resistant depression [9] and recurrent major depressive disorder [10], reduction in Obsessive—Compulsive Disorder symptoms [11], and improvement in addictive behaviors [12,13]. These studies did not note any serious adverse effects. However, one study reported that psilocybin in high doses was at times associated with transient anxiety in some participants.

Hallucinogens are often used as club drugs amongst young Americans because they lead to “trips.” Hollister defined hallucinogens as substances that mainly cause mood, thought, and perception alterations with minimal impairment in memory and intellect. Additionally, they should cause little cravings. Psilocybin, mescaline, and Lysergic acid diethylamide (LSD) are some commonly used classical hallucinogens. 3,4-methylene-deoxy-methamphetamine (MDMA) and phencyclidine (PCP) are other drugs popular among today’s youth; these produce effects similar to classical hallucinogens [14].

When used recreationally, hallucinogens are observed to have a wide variation in psychological and physical effects. Some adverse psychological effects are depersonalization, derealization, paranoia, suicidal ideation, changes in perception, and affective states. MDMA use in the long term can cause many comorbidities in adolescents, including increasing the risk of a hallucinogen use disorder, possible memory deficits in the future, and worsening depressive symptoms [14]. Suicide attempts among adolescents aged 12–17 years who had lifetime MDMA use were found to be close to double of the matched adolescent drug users other than MDMA and close to 9 times for controls who did not use drugs [15].

While much has been published about drugs such as cannabis, alcohol, and nicotine and their association with psychiatric diseases, very few studies have been done on hallucinogens and their association with psychiatric illnesses. One study found a decreased likelihood of psychological distress and suicidality with classic psychedelic use [16]. Jones et al. found a decreased risk of depression in MDMA/ecstasy and psilocybin users [17]. Another found no significant association between mental illness and lifetime use of hallucinogens. [18]. Nesvag et al. disputed this study for selection bias and over-adjustment. Anderson et al. noted improved mood and focus in their study respondents who micro dosed on psychedelics. Though this study suggests the need for more research on micro dosing, recruitment was through an online forum of users of LSD, psilocybin, or both. This study had limitations of online recruitment which is not generalizable to the general population and a small sample size [19]. Shalit et al. found that hallucinogen use was significantly associated with mood disorders, anxiety disorders, eating disorders, personality disorders, and other substance use disorders [20]. In addition, Han et al. found that past year illicit LSD users had a greater risk of serious psychological distress, any/serious mental illness, and suicidal ideation [21]. It is noteworthy that these studies were all done on adults. A study done by Greydanus and Patel in 2003 on substance abuse in adolescents is now outdated [22]. Polysubstance use is a common occurrence, and though a study was done on different illicit drugs and their association with psychiatric disorders, they grouped stimulants and hallucinogens into one class [23]. Mixed findings evident in the past studies on adults call for more studies addressing the risk of hallucinogen use in adolescents, its association with mental illness, and substance use. This paper aimed to primarily evaluate the prevalence and trend of hallucinogen use among US adolescents. Secondarily, we aimed to evaluate the prevalence and odds of hopelessness, suicidality, and co-occurring substance use among adolescent hallucinogen users.

## 2. Materials and Methods

### 2.1. Details of Data

The Youth Risk Behavior Surveillance System (YRBSS) contains data collected by the Centers for Disease Control and Prevention (CDC) to track health behaviors that contribute to the leading causes of morbidity and mortality among youth and adults. YRBSS monitors six major categories: (1) behaviors contributing to violence and unintentional injury, (2) tobacco use, (3) alcohol use and other substance use, (4) risky sexual behaviors associated with unintended pregnancy and sexually transmitted diseases such as HIV infection, (5) dietary habits, and (6) physical inactivity. The YRBSS contains the prevalence of asthma, obesity, and other health issues. To generate a nationally representative sample of 9th to 12th-grade students, YRBSS employs a three-stage cluster sample design. Every two years, a new YRBSS database is released. For our study, we used YRBSS 2001–2019 data.

Further details of YRBSS data are available on the following webpage: https://www.cdc.gov/healthyyouth/data/yrbs/index.htm (accessed on 1 May 2022).

### 2.2. Study Population

Our study population consisted of 125,550 school-going adolescents included in the YRBSS 2001–2019 data. We excluded participants with missing sociodemographic data and substance use data from our analysis.

### 2.3. Outcomes

We primarily aimed to evaluate the prevalence and trends of hallucinogen use among school-going American adolescents. Secondarily, we aimed to identify the prevalence of hopelessness, suicidality, and co-occurring substance use among adolescent hallucinogen users. The tertiary objective is to identify the association between hopelessness, suicidality, and hallucinogen use among US adolescents.

### 2.4. Hallucinogen Use

We identified hallucinogen use based on the following survey question: “During your life, how many times have you used hallucinogenic drugs, such as LSD, acid, PCP, angel dust, mescaline, or mushrooms?” Responses were dichotomized to represent participants who used hallucinogens in the past and participants who never used hallucinogens.

### 2.5. Hopelessness and Suicidality

In order to assess for hopelessness and suicidality in participants, we used the following question from the YRBSS database: (1) Sad or hopeless: “During the past 12 months, did you ever feel so sad or hopeless almost every day for two weeks or more in a row that you stopped doing some usual activities?”. (2) Considered suicide: “During the past 12 months, did you ever seriously consider attempting suicide?”. (3) Made suicide plan: “During the past 12 months, did you make a plan about how you would attempt suicide?”. (4) Attempted suicide: “During the past 12 months, how many times did you actually attempt suicide?”. (5) Injurious suicide attempt: “If you attempted suicide during the past 12 months, did any attempt result in an injury, poisoning, or overdose that had to be treated by a doctor or nurse?”. Responses to all questions were dichotomized.

### 2.6. Co-Occurring Substance Use

To identify the use of other substances, we utilized the following questions from the database: (1) Traditional cigarette: “Have you ever tried cigarette smoking, even one or two puffs?”. (2) Electronic vapor product (e-cigarette): “Have you ever used an electronic vapor product?”. (3) Synthetic marijuana: “During your life, how many times have you used synthetic marijuana?”. (4) Cocaine: “During your life, how many times have you used any form of cocaine, including powder, crack, or freebase?”. (5) Inhalants: “During your life, how many times have you sniffed glue, breathed the contents of aerosol spray cans, or inhaled any paints or sprays to get high?”. (6) Heroin: “During your life, how many times have you used heroin (also called smack, junk, or China White)?”. (7) Methamphetamine: “During your life, how many times have you used methamphetamines (also called speed, crystal meth, crank, ice, or meth)?”. (8) Ecstasy: “During your life, how many times have you used ecstasy (also called MDMA)?”. (9) Alcohol: “How old were you when you had your first drink of alcohol other than a few sips?”. Responses to all questions were dichotomized.

### 2.7. Covariates and Confounders

We accounted for sociodemographic variables and concurrent substance use related variables as potential covariates and confounders in our analysis.

### 2.8. Statistical Analysis

Statistical analysis was performed using IBM SPSS, version 26. The complex sample add-on in SPSS was used to account for three stage cluster design of YRBSS. In descriptive statistics, the Rao-Scott chi-square test was used for categorical variables. Multivariable logistic regression analysis was performed to identify the odds of hopelessness and suicidality among adolescent hallucinogen users after adjusting for previously defined covariates and confounders. 2-sided tests were used. A *p*-value at the level of <0.05 was considered statistically significant. The c-index for goodness of fit for the regression model was calculated.

## 3. Results

### 3.1. Epidemiological Characteristics and Trend of Hallucinogen Use

**Figure 1:**
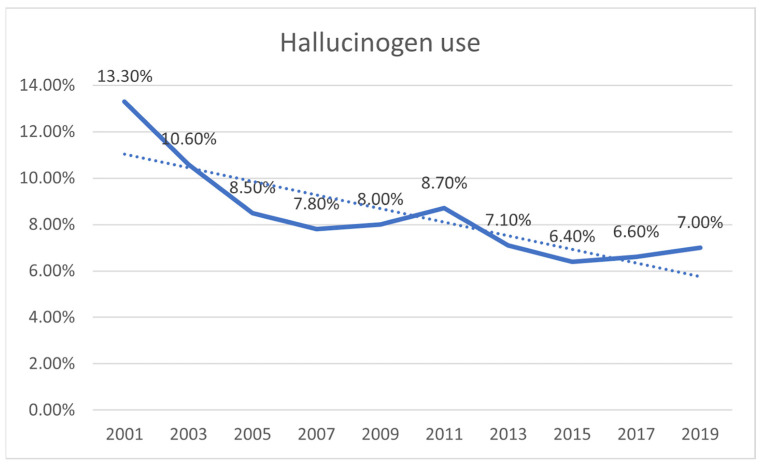
The trend of hallucinogen use among US adolescents 2001–2019. Dotted line represents the trendline.

Out of 125,550 respondents, 8.4% reported using hallucinogens. Overall, the trend of hallucinogen use decreased from 13.3% (2001) to 7.0% (2019) (pTrend < 0.0001) (Figure 1). A higher percentage of male participants reported hallucinogen use than females (10.3% vs. 6.3%; *p* < 0.0001). Among Caucasians, a higher percentage reported hallucinogen use than no use (67.7% vs. 59.7%; *p* < 0.0001). Among Hispanics/Latinos, a slightly higher percentage reported hallucinogen use than no use (19% vs. 18.7%; *p* < 0.0001) (Table 1). Among other races, a less percentage of adolescents reported hallucinogen use compared to no hallucinogen use.

### 3.2. Prevalence of Hopelessness, Suicidality, and Co-Occurring Substance Use among Adolescent Hallucinogen Users

**Figure 2:**
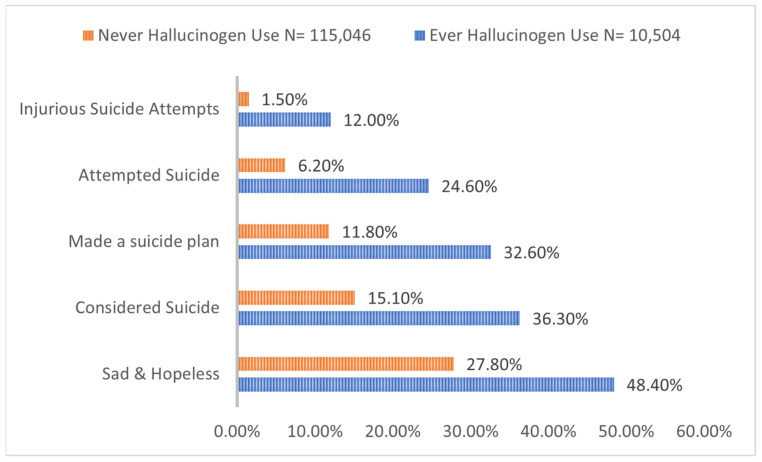
Prevalence of hopelessness and suicidality in adolescent hallucinogen users.

**Figure 3:**
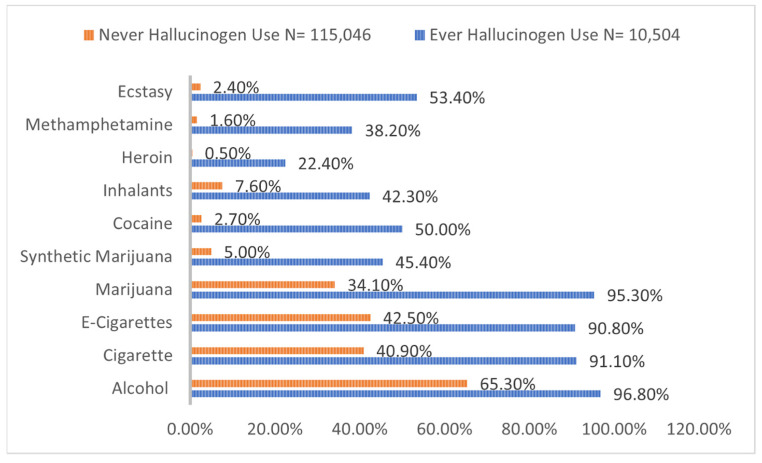
Prevalence of co-occurring substance use in adolescent hallucinogen users.

Compared to adolescents without hallucinogen use, adolescents with hallucinogen use were found to have a higher prevalence of feeling hopeless (48.4 vs. 27.8%), considering suicide (36.3% vs. 15.1%), and attempting suicide that required medical attention (12.0 vs. 1.5%) (*p* < 0.0001 for all) (Figure 2). Additionally, adolescent hallucinogen users had a higher prevalence of alcohol, cigarette, e-cigarette, marijuana, synthetic marijuana, inhalants, heroin, cocaine, methamphetamine, and ecstasy use (Figure 3).

### 3.3. Multivariable Logistic Regression Analysis

In regression, hallucinogen users were at high odds of feeling sad and hopeless (aOR: 1.40; 95%CI: 1.21–1.61; *p* < 0.0001), considering suicide (1.36; 1.08–1.70; *p* = 0.009), and planning suicide (1.49; 1.19–1.86; *p* = 0.001) (Table 2).

## 4. Discussion

We presented data on epidemiology and psychiatric comorbidity in the US adolescent population with hallucinogen use. Our retrospective cross-sectional study found a decreasing trend in hallucinogen use amongst adolescents from 2007 to 2019. Similar to the results of our study, Livne et al. also reported a decrease in trends in adolescent hallucinogen use from the year 2002 to 2019 based on data from the US National Survey on Drug Use and Health [24]. In our study, we found that the prevalence of hallucinogen use was found to be higher in males than in females. We believe that this could be a result of societal response and stigmatization of female drug abusers, differences in physiologic effects, and different personality influences like harm avoidance [25,26]. Similar to results of our study, Lev-Ran reported a higher prevalence of substance use disorder in males with lifetime exposure to hallucinogens than in females [27].

We found a higher prevalence of feeling hopeless, considering, and attempting suicide in adolescent hallucinogen users with high odds of feeling sad and hopeless, considering, and planning suicide. There are a number of studies that have found similar associations between hallucinogen use and suicidality. For example, Shalit et al. found that hallucinogen use was significantly associated with psychiatric disorders such as mood disorders, anxiety disorder, other substance use disorders, and history of past suicide attempts in the US adult population [20]. In our study, we found a statistically significant association between hallucinogen use and suicidal ideation/plan, but results for suicide attempt were not statistically significant. One explanation for such an association could be due to hallucinogen induced altered state of mind. For example, recreational hallucinogens were found to be associated with prolonged psychosis, lasting perceptual abnormalities, dangerous behavior in unsafe settings, and bad trips leading to a challenging experience [4,28]. Hallucinogen persisting perception disorder, as a result of hallucinogen use, has been reported to lead to depression and suicide [29]. A survey study showed that 11% of participants put themselves or others at risk of harm after consuming psilocybin mushrooms [30]. There have been reports of suicide, jumping from a building, and auto-mutilation after hallucinogen use [31,32,33].

Contrary to our study, Johansen et al. did not find a statistically significant association between lifetime use of classical psychedelics and increased likelihood of mental health problems including suicidality [18]. A statistically significant association was found between having used a classical psychedelic in the past, especially psilocybin, and decreased likelihood of psychological distress and suicidality [34]. However, these findings were observed in the adult population of the United States. Thus, there remains a possibility that hallucinogen use may have differential effects on developing brains of adolescents. Results of our study once again highlight the need for more research regarding the effects of hallucinogen use among the adolescent population.

Hallucinogens have indeed shown benefits in a supportive and controlled environment that includes reduction in depressive symptoms, anxiety, and addictive behaviors [35,36,37]. Hallucinogens have also shown benefits in treatment of PTSD [38]. Such benefits could be attributed to their effects on serotonin 5HT2a, 5HT2c, 5HT1a, dopaminergic, adrenergic receptors, and activation of TAAR1 [39,40]. One case series has even described sustained benefits in psychiatric symptoms in participants following LSD overdose; however, due to the case study design, such evidence is not indicative of beneficial effects of hallucinogens in recreational users [41]. We also found a higher prevalence of co-occurring substance use in adolescent hallucinogen users, while it remains to be studied if hallucinogens would increase the risk of using other substances through some specific mechanism. Once of the explanations could be that hallucinogen users may be more induced to experiment with other substances as a result of their personality traits, as well as resultant brain changes secondary to recreational hallucinogen use [42,43].

In summary, we found a high prevalence of hopelessness, suicidality, and co-occurring substance use among adolescent hallucinogen users. Due to the cross-sectional nature of our study, it was not possible to identify the direction of this association. It is possible that hallucinogen use could lead to hopelessness and suicidal ideation among the adolescent population. At the same time, there remains a possibility that depressed and suicidal adolescents may use hallucinogens as a form of self-medication/coping. We highlight the need for further research in exploring the effects of both recreational and therapeutic hallucinogen use among the adolescent population. We recommend longitudinal studies to identify causality between hallucinogen use, hopelessness, and suicidality in the adolescent population.

The strength of our study is its large sample size spanning across years, which provides detailed trend information about adolescent hallucinogen use and results have higher generalizability. Despite these strengths, our study has some limitations. First, the population universe of YRBSS data does not include hallucinogen users who were not enrolled in high school; thus, participants who may have dropped out of high school or were never enrolled would have been missed. Second, although the YRBSS survey questions have good test-retest reliability, we cannot exclude the underreporting or overreporting of health-related behaviors. Third, due to the cross-sectional nature of our study, we can only identify the association, not the causation. Fourth, the YRBSS asks questions about hallucinogen use as a whole while not asking for each individual hallucinogen drug; thus, it was not possible to evaluate the strength and direction of association for each individual hallucinogen. Lastly, even after adjusting for the most common confounders, residual confounding is always present.

## 5. Conclusions

We found an overall decreasing trend of hallucinogen use among American adolescents. We found a high prevalence of hopelessness, suicidality, and co-occurring substance use among hallucinogen users. In an adjusted regression, we found that hallucinogen users were at high odds of feeling sad, and hopeless, and considering and planning suicide. However, we did not find an association between hallucinogen use and suicide attempts. The cross-sectional nature of our study can only identify association, not causation. Further research is needed to explore the effects of recreational hallucinogen use among the adolescent population.

## Figures and Tables

**Table 1:** Epidemiological characteristics of school-going US adolescents—YRBSS 2001–2019.

	Ever Hallucinogen Use *n* = 10,504 (8.4%)	Never Hallucinogen Use *n* = 115,046 (91.6%)
Sex **		
Female	37.6% (9812)	50.5% (57,814)
Male	62.4% (6505)	49.5% (56,763)
Age **		
≤12	1.3% (134)	0.0% (190)
13	0.1% (12)	0.1% (95)
14	6.4% (670)	11.4% (13,016)
15	19.2% (2005)	25.8% (29,613)
16	25.8% (2694)	25.6% (29,370)
17	28.3% (2953)	23.5% (25,885)
≥18	18.9% (1971)	13.6% (15,526)
Race **		
White	67.7% (6968)	59.7% (67,606)
African American	5.0% (511)	13.2% (14,945)
Hispanic/Latino	19.0% (1952)	18.7% (21,170)
All other races	8.4% (863)	8.4% (9493)

% is column percentage, describing the comparison between adolescents with hallucinogen use and no hallucinogen use. Frequency is weighted. ** *p* < 0.0001.

**Table 2:** Multivariable logistic regression analysis for the association between hallucinogen use, and hopelessness and suicidality among American adolescents.

	Adjusted Odds Ratio	95% Confidence Interval	*p*-Value
Sad & Hopeless (a)	1.38	1.19–1.60	<0.0001
Considered Suicide (b)	1.31	1.02–1.69	0.03
Made Suicide Plan (c)	1.44	1.13–1.83	0.003
Attempted Suicide (d)	1.15	0.83–1.61	0.395
Injurious Suicide Attempt (d)	1.39	0.92–2.11	0.118

(a) Adjusted for age, sex, race, co-occurring substance use, (b) Adjusted for (a) and feeling sad and hopeless, (c) Adjusted for (b) and considering suicide, (d) Adjusted for (c) and making a suicide plan.

## Data Availability

The data that support the findings of this study are openly available in the Youth Risk Behavior Surveillance System at https://www.cdc.gov/healthyyouth/data/yrbs/index.htm (accessed on 30 October 2022). Software and code is available upon request.
